# Current Strategies to Inhibit High Affinity FcεRI-Mediated Signaling for the Treatment of Allergic Disease

**DOI:** 10.3389/fimmu.2019.00175

**Published:** 2019-02-07

**Authors:** Gregorio Gomez

**Affiliations:** Department of Pathology, Microbiology and Immunology, University of South Carolina School of Medicine, Columbia, SC, United States

**Keywords:** FcεRI, allergy, omalizumab, DARPin, fusion protein, mast cells, basophils, FcγRIIb

## Abstract

Allergies and asthma are a major cause of chronic disease whose prevalence has been on the rise. Allergic disease including seasonal rhinitis, atopic dermatitis, urticaria, anaphylaxis, and asthma, are associated with activation of tissue-resident mast cells and circulating basophils. Although these cells can be activated in different ways, allergic reactions are normally associated with the crosslinking of the high affinity Fc receptor for Immunoglobulin E, FcεRI, with multivalent antigen. Inflammatory mediators released from cytoplasmic granules, or biosynthesized *de novo*, following FcεRI crosslinking induce immediate hypersensitivity reactions, including life-threatening anaphylaxis, and contribute to prolonged inflammation leading to chronic diseases like asthma. Thus, inappropriate or unregulated activation of mast cells and basophils through antigenic crosslinking of FcεRI can have deleterious, sometimes deadly, consequences. Accordingly, FcεRI has emerged as a viable target for the development of biologics that act to inhibit or attenuate the activation of mast cells and basophils. At the forefront of these strategies are (1) Anti-IgE monoclonal antibody, namely omalizumab, which has the secondary effect of reducing FcεRI surface expression, (2) Designed Ankyrin Repeat Proteins (DARPins), which take advantage of the most common structural motifs in nature involved in protein-protein interactions, to inhibit FcεRI-IgE interactions, and (3) Fusion proteins to co-aggregate FcεRI with the inhibitory FcγRIIb. This review presents the published research studies that support omalizumab, DARPins, and fusion proteins as, arguably, the three most currently viable strategies for inhibiting the expression and activation of the high affinity FcεRI on mast cells and basophils.

## Introduction

Allergic disease refers to a variety of disorders that include seasonal allergies, atopic dermatitis, urticaria, life-threatening anaphylaxis reactions to food, and allergic asthma. Curiously, the incidence of allergic disease has increased dramatically in recent decades, and continues to rise in developed countries. Allergies and asthma are among the most prevalent chronic diseases worldwide (1, 2). The culprits are a variety of pre-formed inflammatory mediators including histamine, serine proteases, proteoglycans, and other enzymes, that are stored in cytoplasmic granules and released from mast cells and basophils immediately following “degranulation,” and eicosanoids like prostaglandins and leukotrienes that are very rapidly biosynthesized from arachidonic acid. Prolonged stimulation also induces the activation of various transcription factors, and synthesis of new cytokines that contribute to inflammation and recruitment of other cell types.

Mast cells can be activated by a variety of agents. However, allergic reactions are generally associated with crosslinking of the high affinity Fc receptor for immunoglobulin E (IgE), FcεRI, with multivalent antigen (3). High affinity FcεRI is comprised of an IgE-binding α chain, a signal enhancing β chain, and two signal transducing γ chains. The tetrameric receptor, α*βγ*2, is expressed predominantly on tissue-resident mast cells and circulating basophils (4). However, in a proportion of human subjects, mostly atopic patients, a trimeric form of the receptor lacking the β chain, αγ2, is expressed on other cell types including airway smooth muscle (5), bronchial and intestinal epithelial cells (6, 7), Langerhan cells (8, 9), dendritic cells (10, 11), monocytes (12), and eosinophils (13), neutrophils and platelets (14–16).

Binding of IgE to FcεRI on mast cells and basophils enhances FcεRI expression (17–21). It is thought that IgE binding to FcεRI protects the receptor from being internalized and degraded. On the other hand, IgE binding to FcεRI on dendritic cells and monocytes (but not basophils) facilitates the internalization and degradation of IgE-bound FcεRI within endolysosomal compartments (22). In addition to showing that IgE levels are important in stabilizing FcεRI expression, these observations also indicate a role for FcεRI in clearance of serum IgE. Moreover, they suggest that α*βγ*2 expressed on mast cells and basophils is predominantly involved in signal transduction leading to mast cell and basophil activation or degranulation, whereas αγ2 on antigen presenting cells is mostly involved in IgE-FcεRI internalization.

**Figure 1:**
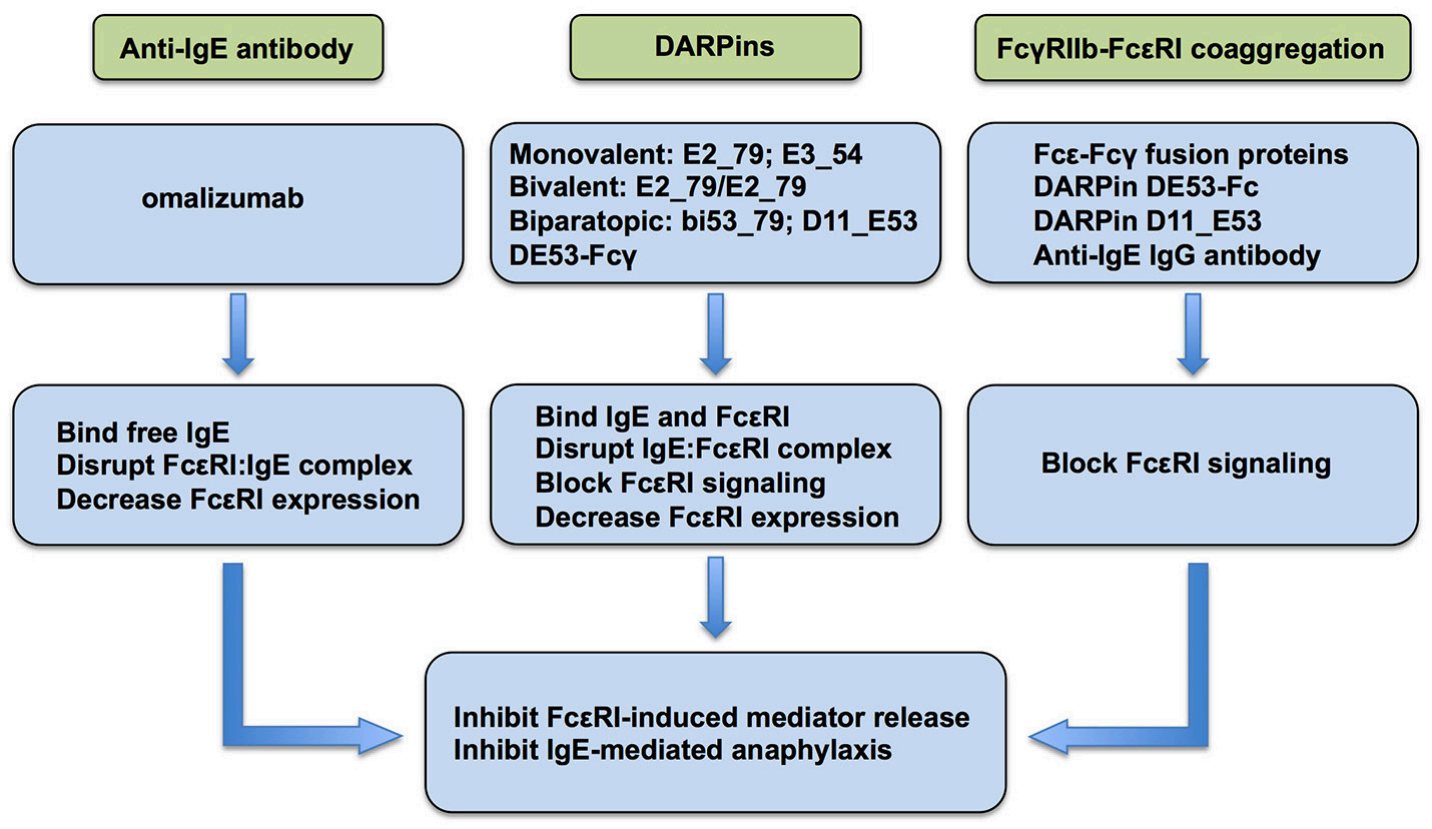
Current strategies to inhibit FcεRI signaling in allergic disease.

The role of FcεRI as the primary activator of mast cells and basophils leading to the release of allergic/inflammatory mediators resulting in IgE-mediated immediate hypersensitivity reactions and allergic inflammation is well-documented (3). Accordingly, FcεRI has emerged as a target of biologics for regulating allergic reactions. Currently, anti-IgE monoclonal antibody omalizumab, DARPins, and fusion proteins that co-aggregate FcεRI and FcγRIIb are at the forefront of the strategies currently employed or actively being investigated as a means of regulating the expression and/or activation of FcεRI for the therapeutic purpose of inhibiting mast cells and basophils (Figure 1).

## Omalizumab

Perhaps the most studied strategy directed against allergic disease is the use of anti-IgE antibodies. Omalizumab (Xolair®) is a humanized anti-IgE mouse monoclonal antibody that is FDA-approved for the treatment of mild to severe allergic asthma and chronic spontaneous urticaria (23–26). Omalizumab works by binding to circulating free IgE, thereby, reducing the amount that would normally be available to bind FcεRI on mast cells and basophils. In an early Phase I study of 15 allergic and asthmatic patients with serum levels of IgE between 187 and 1,210 ng/ml, intravenous injection of omalizumab resulted in reduction of IgE to 1% of pre-treatment levels (27). It is widely reported that omalizumab competes with FcεRI for the C3ε domain of IgE, thus preventing it from binding FcεRI-bound IgE (28, 29). However, another study reported that steric hindrance by C2ε domain, rather than direct competition for site binding, was responsible for the inability of omalizumab to bind FcεRI-bound IgE (30). Regardless, omalizumab cannot bind IgE bound to FcεRI on mast cells or basophils, and, therefore, does not crosslink IgE-bound FcεRI to induce the release of allergic mediators. Since binding of IgE to FcεRI on mast cells and basophils enhances the expression of FcεRI (17–21), the reduction in free IgE by omalizumab leads to diminished expression of FcεRI on the surface of mast cells, basophils, and dendritic cells (21, 27, 31, 32). In one study, treatment of atopic individuals with omalizumab for 3 months reduced the expression of FcεRI on basophils by ~97% from ~220,000 to ~8,300 receptors per basophil (27). An *in vitro* study with *in situ*-matured mast cells from human skin demonstrated that IgE-dependent enhancement of FcεRI on human skin mast cells was both prevented and reversed by omalizumab (21). In this study, omalizumab prevented the upregulation of FcεRI by 90% when added simultaneously with polyclonal IgE at a molar ratio of 2.9 (omalizumab to IgE). Omalizumab also dose-dependently decreased FcεRI expression on human skin mast cells when added to cultures after FcεRI had already been upregulated with IgE, suggesting that omalizumab could disassemble pre-formed IgE:FcεRI complexes. This was later confirmed with a cell-free system and human basophils (30, 33). The exact mechanism by which omalizumab “strips” IgE off of FcεRI is not exactly known, but allosteric destabilization and facilitated dissociation of the IgE:FcεRI complex, at least at high concentrations of omalizumab, are suspected (33–36). Human skin mast cells with IgE-enhanced FcεRI levels were more sensitive to stimulation with a low dose of anti-FcεRI mAb compared to mast cells with basal levels of FcεRI in terms of degranulation, PGD_2_ biosynthesis, and cytokine production. Reduction of FcεRI levels with omalizumab restored sensitivity to stimulation, and mediator release, to basal levels.

The efficacy and safety of omalizumab as treatment against allergic asthma and urticaria has clearly been demonstrated, including as an add-on therapy with traditional treatments such as glucocorticoids (23, 24). The therapeutic potential of omalizumab in other IgE-mediated disorders in which FcεRI plays a role, including food allergy (37–39), allergic rhinitis (40, 41), and atopic dermatitis (42, 43) has also been demonstrated. However, one major concern is the duration of the positive effects of omalizumab post-treatment. In one study (44), serum free IgE was reduced by 96–98%, and wheal-and-flare reactions to skin prick tests were significantly reduced in 40 patients with allergic rhinitis who were treated with omalizumb for 28 weeks. However, serum free IgE levels and skin reactivity increased following a reduction in the amount of omalizumab administered, and returned to baseline when therapy was completely discontinued. In another study (45), loss of control of asthma symptoms following discontinuation of omalizumab was recorded in 57% of the participants with a median time-point of 13 months after discontinuation. In these studies, FcεRI levels on mast cells or basophils was not monitored, but given that omalizumab decreases FcεRI expression on these cell types (21, 27, 31, 32), it is expected that receptor expression increased when treatment was terminated. Thus, treatment with omalizumab could require personalized optimization in terms of dosage and duration of treatment to yield maximal benefits.

Omalizumab as an adjunct to allergen immunotherapy (AIT) against IgE-mediated food allergy and allergic asthma is also currently under investigation (46–50). The main types of AIT are subcutaneous immunotherapy (SCIT) and sublinguinal immunotherapy (SLIT) (51). SCIT and SLIT have been shown to be efficacious for perennial and seasonal allergic respiratory disease (50, 52, 53). However, SCIT or SLIT are contraindicated for severe or uncontrolled asthma (54). It is thought that pre-treatment with omalizumab of patients with severe uncontrolled asthma, which has been shown to be efficacious, could allow AIT in patients that previously could not tolerate it (48, 55). However, studies to investigate AIT in combination with omalizumab are currently lacking. With regard to food allergies, omalizumab treatment in conjunction with oral immunotherapy (OIT) has shown promise in desensitizing allergic patients to peanuts, milk, and multiple food allergens (56–60). Overall, the few reported studies have shown promise for the use of omalizumab in combination with AIT for IgE-mediated disease.

Other anti-IgE antibodies have also been developed and tested including Ligelizumab (QGE031), Quilizumab (MEMP1972A), XmAb7195, and MEDI4212 that might provide additional opportunities for anti-IgE therapy in the future (61). To date, however, none have been shown to be clinically superior to omalizumab, or data is still coming out. In some cases, for example QGE031 for asthma, development has been discontinued. Nevertheless, these or other anti-IgE antibodies could provide additional opportunities for anti-IgE therapy in the future.

## DARPins

DARPins (designed ankyrin repeat proteins) are a class of small (14–21 kDa) binding proteins comprised of a varying number of stacked ankyrin repeat domains (62), which are one of the most common structural motifs involved in protein-protein interactions in nature. Natural ankyrin repeats are 33 residue motifs comprised of two α-helical structures connected by a loop that stack one on top of the other to form ankyrin repeat domains (63). A single DARPin library module is comprised of a 33 residue repeat of which seven residues are randomized and non-conserved. Typically, two to four library modules are genetically fused and flanked by N-cap and C-cap repeats to form one protein domain (64, 65). Binding of ankyrin repeat domains can affect stability and effector function of the target protein. The motivation for engineering DARPins was to generate binding proteins that could be used to target proteins with high affinity and specificity, essentially replacing the use of monoclonal antibodies (62).

In one of the first studies (66), two monovalent DARPins (B-A4-85 and C-A3-30) capable of binding two different epitopes of human FcεRIα were identified and successfully fused to each other with the flexible linker [Gly_4_-Ser]_4_. A bispecific DARPin (30/85) was identified as being capable of simultaneously binding FcεRIα at both epitopes with affinity for FcεRIα greater than that of IgE. In *in vitro* studies, DARPin 30/85 blocked IgE binding to FcεRI, and inhibited IgE-induced degranulation of human FcεRIα-transfected RBL-2H3 cells to a similar extent as omalizumab. In a similar study (67), two monovalent DARPins, E2_79 and E3_54, that were specific for IgE, and could inhibit IgE-FcεRI interactions, were identified. Bivalent proteins were genetically engineered by coupling the monovalent DARPins with the glycine-serine linker. E2_79/E2_79, at 5-fold molar excess with IgE, inhibited the binding of IgE to FcεRIα by >90%, comparable binding by omalizumab. E2_79/E2_79 also effectively bound free IgE in serum. The researchers further demonstrated that both the monovalent and bivalent DARPins inhibited IgE-mediated degranulation of FcεRIα-transfected RBL-2H3 cells. Bivalent DARPin E2_79/E2_79 was particularly effective, exhibiting an IC_50_ of 0.54 nM compared to 1.77 nM for omalizumab. It was later shown that E2_79, in addition to binding free IgE, could also stimulate the dissociation of pre-formed IgE:FcεRI complexes by a facilitated dissociation mechanism at one of two binding sites identified for E2_79 on the IgE:FcεRI complex (36). In a separate study, treatment with E2_79 significantly reduced surface expression of FcεRI on human *ex vivo* isolated primary basophils, and inhibited FcεRI-induced activation and leukotriene C4 (LTC_4_) biosynthesis (30). Further, a biparatopic DARPin, bi53_79, which was engineered by fusing the disruptive E2_79 with non-disruptive E3_53 anti-IgE DARPins exhibited a >10-fold increase in capacity to disrupt FcεRI:IgE complexes, and was more effective at inhibiting anaphylactic reactions *in vivo* compared with E3_79 alone. Noteworthy, E2_79 and bi53_79 acted faster and were more effective than omalizumab in parallel experiments. These studies demonstrate the therapeutic potential of DARPins as inhibitors of FcεRI-induced allergic reactions. Thus, supporting the notion that DARPins have the potential to supplant monoclonal antibodies such as omalizumab as treatment for allergic asthma and other allergic diseases (62, 65).

However, DARPins are protein structures, and the potential for immunoreactivity resulting from the production of anti-DARPin antibodies should be met with extreme caution. Clearly the immune response to DARPin proteins could be a major limitation in the use of DARPins as therapeutic agents. In addition, the possibility of negative or deleterious effects of inhibiting the activation of FcεRI-expressing cell types should also be considered. For example, mast cells and eosinophils play a major role in the clearance and expulsion of parasites particularly helminths. Likewise, mast cell mediators also protect against insect and reptile venom. Thus, blocking the activation of mast cells could inhibit the positive or protective effects associated with FcεRI activation. This might be particularly relevant in countries where parasitic infections are endemic. It is argued that DARPins would be more cost effective than monoclonal antibodies because they can be produced in large scale in bacteria; however, the relative cost to human safety must be considered. Importantly, in July 2018, Allergan and Molecular Partners announced that Abicipar pegol, a DARPin engineered to target vascular endothelial growth factor (VEGF), had reached the primary end point in two Phase III trials for the treatment of neovascular age-related macular degeneration (AMD). In two trials, Abicipar pegol demonstrated non-inferiority to the approved anti-VEGF ranibizumab (Lucentis®). Of significant concern, however, was a significantly greater incidence of ocular inflammation with Abicipar pegol than Lucentis®. Allergan is expected to file Abicipar pegol with the FDA in early 2019. Thus, whether DARPins are safe and efficacious in humans is currently being determined.

## Co-aggregation of FcεRI With FcγRIIb

Given the requirement for tyrosine phosphorylation events in the initiation and propagation of FcεRI signaling in mast cells and basophils (68–72), one strategy to inhibit FcεRI-mediated reactions has been to take advantage of the inhibitory property of FcγRIIb. FcγRIIb is the only known inhibitory IgG Fc receptor (73, 74). In contrast to FcεRI, which utilizes immunoreceptor tyrosine-based activation motif (ITAM), FcγRIIb utilizes the inhibitory counterpart (ITIM) that, upon receptor activation, recruits SH2-domain containing phosphatases including SHIP. The phosphatases interfere with the tyrosine-based activation of early signaling molecules resulting in the inhibition of signal transduction (75–77). FcγRIIb is expressed on human basophils and cord blood-derived mast cells (78–80). It is not constitutively expressed on human skin mast cells (81), but FcγRIIb expression can be induced in human intestinal mast cells with interferon γ (82) and on human basophils with IL-3 (79) suggesting that it could be induced in tissue-derived mast cells. Various experiments have been performed demonstrating that co-aggregation of FcεRI and FcγRIIb inhibits IgE-dependent activation and mediator release from mast cells and basophils. In one study (83), it was demonstrated that serotonin release from mouse bone marrow-derived mast cells (BMMCs) sensitized with anti-ova IgE, and then challenged with ova, was dose-dependently inhibited when the BMMCs were challenged with DNP-ova complexed with anti-DNP IgG. The requirement for co-aggregation of FcεRI and FcγRIIb to inhibit mast cell mediator release was further tested and confirmed in rat basophilic leukemia cells (RBL-2H3) transfected with FcγRIIb. Another study (84) used a bispecific antibody expressing one Fab fragment specific for human IgE, and the other for FcγRIIb, to show that simultaneous crosslinking of FcεRI and FcγRIIb inhibited antigen induced histamine release from human cord blood-derived mast cells and peripheral blood basophils. Cassard et al. (79) used an IgG anti-IgE, which binds FcεRI-bound IgE via its Fab, and FcγR via their Fc domain, to demonstrate that co-aggregation of FcεRI and FcγRIIb negatively regulates IgE-induced activation of human and mouse basophils, and release of histamine and IL-4. Furthermore, a comprehensive *in vivo* study utilizing passive and active immunization of mice determined that FcεRI-FcγRIIb crosslinking contributed significantly to the inhibition of IgE-mediated anaphylaxis by IgG blocking antibodies particularly under low concentrations of IgG blocking antibody (85). Collectively, these studies support the notion that co-aggregation of FcεRI and FcεRIIb is a viable strategy to limit allergic responses.

Over the years, Fcε-Fcγ fusion proteins to co-aggregate FcεRI and FcγRIIb have been investigated. One of the earliest bi-functional fusion proteins that was engineered, termed GE2, is comprised of the hinge-Cγ2-Cγ3 domains of the human IgG Fc and Cε2-Cε4 domains of human IgE Fc connected by a 15 amino acid (Gly_4_-Ser)_3_ linker (86). Human GE2 was shown to bind to both FcεRI and FcγRII at levels equivalent to human IgE and IgG, respectively. Functionally, GE2 inhibited IgE-dependent degranulation of human basophils in time- and dose-dependent manner with maximal inhibition observed when the cells were sensitized with antigen-specific IgE and GE2 simultaneously. GE2 co-aggregation of FcεRI and FcγRII inhibited Syk phosphorylation, a critical event in FcεRI signaling (87, 88), and *in vivo* IgE-induced passive cutaneous anaphylaxis in transgenic mice expressing a human FcεRIα. Kepley, et al. (78) subsequently used GE2 to further demonstrate that co-aggregation of FcεRI and FcγRII on human umbilical cord blood-derived mast cells inhibited degranulation and cytokine production. In a similar study, Mertsching et al. (89) created a murine homolog of human GE2, termed mGE, consisting of Cγ_2a_2-Cγ_2a_3 and Cε2-Cε3-Cε4 domains connected by the (Gly_4_-Ser)_3_ linker. mGE was shown to inhibit IgE-dependent degranulation and cytokine production from wild type but not FcγRIIb-deficient mice BMMCs. mGE also inhibited *in vivo* passive cutaneous and systemic anaphylaxis in mice, with extended protection. Conversely, mGE treatment increased FcγRIIb phosphorylation and its association with SHIP and SHP1/2 phosphatases.

In an effort to enhance the efficacy of FcεRI-FcγRIIb co-engagement while eliminating the possibility of FcεRI crosslinking, Cemerski et al. (90) engineered a tandem Fcε-Fcγ fusion protein comprised of a murine Fcε domain linked to a human Fcγ domain IgG_1_, which, due to S267E and L328F amino acid substitutions at the Fcγ domain, exhibited >100-fold greater affinity for human FcγRIIb compared to the native IgG Fc composition (91, 92). This fusion protein was shown to inhibit IgE-dependent degranulation of human FcγRIIb transgenic BMMCs. However, in the reported experiments, the tandem fusion protein containing the native IgG Fc domain inhibited mast cell degranulation to a similar extent as a control tandem fusion protein lacking affinity for FcγRIIb. The authors concluded that inhibition of mast cell degranulation by co-engagement is more potently suppressed when the tandem fusion protein has higher affinity for FcγRIIb. To our knowledge, the tandem Fc fusion protein with enhanced affinity for FcγRIIb has not been compared to the other reported FcεRI-FcγRII fusion proteins, GE2 (86) and hGE2 (89).

Two pre-clinical studies in non-human primates have demonstrated the potential clinical applicability of FcεRI-FcγRIIb fusion proteins in inhibiting allergic reactions. Zhang et al. (93) first demonstrated that GE2 could inhibit mediator release from mast cells and basophils that had been pre-sensitized with IgE before treatment with GE2 as would be the case in allergic individuals undergoing treatment. The researchers demonstrated that GE2 inhibited Fel d 1 (cat allergen)-induced histamine release from human basophils and lung mast cells from cat allergic patients. Mirroring this, GE2 blocked Fel d 1-induced passive cutaneous anaphylaxis in human FcεRIα transgenic mice that were sensitized with serum from cat allergic subjects. GE2 itself was shown to not induce mediator release or induce anaphylaxis. In their pre-clinical study, GE2 was shown to inhibit skin test reactivity to dust mite (*Dermatophagoides farinae*) allergen in Rhesus monkeys that were naturally allergic to the *D. farina* allergen. In a later study, Mertsching et al. (89) generated another FcεRI-FcγRIIb fusion protein, termed hGE2, based on the GE2 construct of Zhu et al. (86) absent of any non-native sequences. hGE2, administered to cynomolgus monkeys that had been sensitized with the roundworm *Ascaris suum*, was shown to protect the monkeys from cutaneous anaphylaxis induced with *A. suum* extract. The monkeys were reportedly protected from local anaphylaxis for up to three weeks.

Interestingly, a humanized monoclonal anti-IgE antibody (XmAb7195) was reported to have an IgE-binding affinity 5.3-fold greater than omalizumab, and 400 times greater binding affinity for FcγRIIb due to mutations in its Fc region (94). XmAb7195 was shown to block free IgE and inhibit IgE production in B cells by co-engaging IgE and FcγRIIb. Although XmAb7195 did not bind FcεRI-bound IgE (94), this study supports the notion of using anti-IgE IgG antibodies to co-aggregate FcγRIIb and FcεRI to inhibit allergic disease. First-in-Human Phase 1 clinical trials have been conducted with XmAb7195, but results on safety, tolerability and bioavailability have not been reported (61).

DARPins have also been used to co-aggregate FcεRI and FcγRIIb. Eggel et al. (95) generated an anti-IgE DARPin fusion protein in which DARPin E53, which showed reactivity against a non-FcεRIα epitope capable of binding free and receptor-bound IgE, was joined via the (Gly_4_-Ser)_3_ linker to a human IgG_1_ Fc region. DE53-Fc, as it was named, was shown to not be anaphylactogenic, and inhibited allergen-induced activation of basophils in whole blood samples from allergic donors. In a subsequent study (96), a DE53-Fc mutant construct with increased affinity for FcγRIIb due to a single site-directed point mutation in the IgG Fc region was shown to be more efficient at co-aggregating FcεRI and FcγRIIb, resulting in enhanced inhibition of basophil activation. Recently, Zellweger et al. (97) generated DARPin D11_E53, which simultaneously bound human FcγRIIb and FcεRI-bound IgE. The bispecific molecule was shown to inhibit allergen-induced degranulation and LTC_4_ biosynthesis in human primary basophils and huFcεRIα-expressing mouse BMMCs *in vitro*, and decreased *in vivo* passive systemic anaphylaxis induced in huFcεRIα transgenic mice. This study demonstrated that FcγRIIb-mediated inhibition of degranulation requires direct ligation with FcεRI, and that DARPins, at least D11_E53, could safely be applied to animals to inhibit anaphylaxis.

## Concluding Comments

The dramatic increase in prevalence of allergies warrants additional research to develop new strategies and therapies to treat allergic disease. At the forefront are the anti-IgE monoclonal antibody omalizumab, DARPins, and fusion proteins that directly or indirectly alter FcεRI expression and activation. In order to maximize the use of omalizumab, additional clinical studies are needed to identify allergic diseases against which omalizumab could be effective beyond asthma and spontaneous urticaria. The development of newer anti-IgE antibodies could also have an impact. The development of DARPins hold the promise of targeting FcεRI or IgE with greater specificity and better efficacy than monoclonal antibodies without the hurdles associated with development of humanized monoclonal antibodies. As potential clinical therapeutics, DARPins also have the potential to reach a broader population since allotypic differences associated with the use of monoclonal antibodies might not factor in their development. However, safety issues regarding immunogenicity due to anti-DARPin antibodies and unwanted effects due to inhibiting positive effects of mast cell activation must be considered. Whether DARPins can supersede monoclonal antibodies remains to be determined. Harnessing the inhibitory properties of FcγRIIb to inhibit FcεRI with fusion proteins also shows promise as evidenced in pre-clinical studies with non-human primates. It is hoped that these strategies will lead to therapeutics that provide relief to the millions of people worldwide suffering from allergic disease.

## Author Contributions

The author confirms being the sole contributor of this work and has approved it for publication.

### Conflict of Interest Statement

The author declares that the research was conducted in the absence of any commercial or financial relationships that could be construed as a potential conflict of interest.

## References

[1] Wikstén J Toppila-Salmi S Mäkelä M. Primary prevention of airway allergy. Curr Treat Options Allergy (2018) 5:347–55. 10.1007/s40521-018-0190-4 30524932 PMC6244611

[2] Vercelli D. Does epigenetics play a role in human asthma? Allergol Int. (2016) 65:123–6. 10.1016/j.alit.2015.12.001 26778244

[3] Galli SJ Tsai M. IgE and mast cells in allergic disease. Nat Med. (2012) 18:693–704. 10.1038/nm.2755 22561833 PMC3597223

[4] Kraft S Kinet J-P. New developments in FcepsilonRI regulation, function and inhibition. Nat Rev Immunol. (2007) 7:365–78. 10.1038/nri2072 17438574

[5] Gounni AS Wellemans V Yang J Bellesort F Kassiri K Gangloff S. Human airway smooth muscle cells express the high affinity receptor for IgE (Fc epsilon RI): a critical role of Fc epsilon RI in human airway smooth muscle cell function. J Immunol. (2005) 175:2613–21. 10.4049/jimmunol.175.4.2613 16081836

[6] Campbell AM Vachier I Chanez P Vignola AM Lebel B Kochan J. Expression of the high-affinity receptor for IgE on bronchial epithelial cells of asthmatics. Am J Respir Cell Mol Biol. (1998) 19:92–7. 10.1165/ajrcmb.19.1.2648 9651184

[7] Untersmayr E Bises G Starkl P Bevins CL Scheiner O Boltz-Nitulescu G. The high affinity IgE receptor Fc epsilonRI is expressed by human intestinal epithelial cells. PLoS ONE (2010) 5:e9023. 10.1371/journal.pone.0009023 20126404 PMC2814858

[8] Bieber T la Salle de H Wollenberg A Hakimi J Chizzonite R Ring J. Human epidermal Langerhans cells express the high affinity receptor for immunoglobulin E (Fc epsilon RI). J Exp Med. (1992) 175:1285–90. 1533242 10.1084/jem.175.5.1285 PMC2119213

[9] Wang B Rieger A Kilgus O Ochiai K Maurer D Födinger D. Epidermal Langerhans cells from normal human skin bind monomeric IgE via Fc epsilon RI. J Exp Med. (1992) 175:1353–65. 1533243 10.1084/jem.175.5.1353 PMC2119204

[10] Foster B Metcalfe DD Prussin C. Human dendritic cell 1 and dendritic cell 2 subsets express FcepsilonRI: correlation with serum IgE and allergic asthma. J Allergy Clin Immunol. (2003) 112:1132–8. 10.1016/j.jaci.2003.09.011 14657872

[11] Maurer D Fiebiger S Ebner C Reininger B Fischer GF Wichlas S. Peripheral blood dendritic cells express Fc epsilon RI as a complex composed of Fc epsilon RI alpha- and Fc epsilon RI gamma-chains and can use this receptor for IgE-mediated allergen presentation. J Immunol. (1996) 157:607–16. 8752908

[12] Maurer D Fiebiger E Reininger B Wolff-Winiski B Jouvin MH Kilgus O. Expression of functional high affinity immunoglobulin E receptors (Fc epsilon RI) on monocytes of atopic individuals. J Exp Med. (1994) 179:745–50. 8294882 10.1084/jem.179.2.745 PMC2191351

[13] Rajakulasingam K Till S Ying S Humbert M Barkans J Sullivan M. Increased expression of high affinity IgE (FcepsilonRI) receptor-alpha chain mRNA and protein-bearing eosinophils in human allergen-induced atopic asthma. Am J Respir Crit Care Med. (1998) 158:233–40. 10.1164/ajrccm.158.1.9708106 9655735

[14] Hasegawa S Pawankar R Suzuki K Nakahata T Furukawa S Okumura K. Functional expression of the high affinity receptor for IgE (FcepsilonRI) in human platelets and its' intracellular expression in human megakaryocytes. Blood (1999) 93:2543–51. 10194433

[15] Gounni AS Lamkhioued B Koussih L Ra C Renzi PM Hamid Q. Human neutrophils express the high-affinity receptor for immunoglobulin E (Fc epsilon RI): role in asthma. FASEB J. (2001) 15:940–9. 10.1096/fj.00-0378com 11292654

[16] Joseph M Gounni AS Kusnierz JP Vorng H Sarfati M Kinet JP. Expression and functions of the high-affinity IgE receptor on human platelets and megakaryocyte precursors. Eur J Immunol. (1997) 27:2212–8. 10.1002/eji.1830270914 9341761

[17] Furuichi K Rivera J Isersky C. The receptor for immunoglobulin E on rat basophilic leukemia cells: effect of ligand binding on receptor expression. Proc Natl Acad Sci USA. (1985) 82:1522–5. 3156380 10.1073/pnas.82.5.1522 PMC397295

[18] Lantz CS Yamaguchi M Oettgen HC Katona IM Miyajima I Kinet JP. IgE regulates mouse basophil Fc epsilon RI expression *in vivo*. J Immunol. (1997) 158:2517–21. 9058781

[19] Yamaguchi M Lantz CS Oettgen HC Katona IM Fleming T Miyajima I. IgE enhances mouse mast cell Fc(epsilon)RI expression *in vitro* and *in vivo*: evidence for a novel amplification mechanism in IgE-dependent reactions. J Exp Med. (1997) 185:663–72. 9034145 10.1084/jem.185.4.663 PMC2196143

[20] Yamaguchi M Sayama K Yano K Lantz CS Noben-Trauth N Ra C. IgE enhances Fc epsilon receptor I expression and IgE-dependent release of histamine and lipid mediators from human umbilical cord blood-derived mast cells: synergistic effect of IL-4 and IgE on human mast cell Fc epsilon receptor I expression and mediator release. J Immunol. (1999) 162:5455–65. 10228025

[21] Gomez G Jogie-Brahim S Shima M Schwartz LB. Omalizumab reverses the phenotypic and functional effects of IgE-enhanced Fc epsilonRI on human skin mast cells. J Immunol. (2007) 179:1353–61. 10.4049/jimmunol.179.2.1353 17617628 PMC2396781

[22] Greer AM Wu N Putnam AL Woodruff PG Wolters P Kinet JP. Serum IgE clearance is facilitated by human FcεRI internalization. J Clin Invest. (2014) 124:1187–98. 10.1172/JCI68964 24569373 PMC3938266

[23] Israel E Reddel HK. Severe and difficult-to-treat asthma in adults. N Engl J Med. (2017) 377:965–76. 10.1056/NEJMra1608969 28877019

[24] Pelaia C Calabrese C Terracciano R de Blasio F Vatrella A Pelaia G. Omalizumab, the first available antibody for biological treatment of severe asthma: more than a decade of real-life effectiveness. Ther Adv Respir Dis. (2018) 12:1–16. 10.1177/1753466618810192 30400762 PMC6236630

[25] Saini SS Kaplan AP. Chronic spontaneous urticaria: the devil's itch. J Allergy Clin Immunol Pract. (2018) 6:1097–106. 10.1016/j.jaip.2018.04.013 30033911 PMC6061968

[26] Maurer M Rosén K Hsieh HJ Saini S Grattan C Gimenéz-Arnau A Omalizumab for the treatment of chronic idiopathic or spontaneous urticaria. N Engl J Med. (2013) 368:924–35. 10.1056/NEJMoa1215372 23432142

[27] MacGlashan DW Bochner BS Adelman DC Jardieu PM Togias A McKenzie-White J. Down-regulation of Fc(epsilon)RI expression on human basophils during *in vivo* treatment of atopic patients with anti-IgE antibody. J Immunol. (1997) 158:1438–45. 9013989

[28] Qiao CX Lv M Guo LM Yu M Li Y Lin Z. Inhibition of IgE activity to bind its high affinity receptor (FcεRIα) by mouse anti-IgE Cε3**~**4 monoclonal antibody (QME5). Int J Biomed Sci. (2009) 5:336–44. 23675156 PMC3614804

[29] Zheng L Li B Qian W Zhao L Cao Z Shi S. Fine epitope mapping of humanized anti-IgE monoclonal antibody omalizumab. Biochem Biophys Res Commun. (2008) 375:619–22. 10.1016/j.bbrc.2008.08.055 18725193

[30] Eggel A Baravalle G Hobi G Kim B Buschor P Forrer P. Accelerated dissociation of IgE-FcεRI complexes by disruptive inhibitors actively desensitizes allergic effector cells. J Allergy Clin Immunol. (2014) 133:1709–19.e8. 10.1016/j.jaci.2014.02.005 24642143 PMC4083100

[31] Beck LA Marcotte GV Macglashan D Togias A Saini S. Omalizumab-induced reductions in mast cell Fce psilon RI expression and function. J Allergy Clin Immunol. (2004) 114:527–30. 10.1016/j.jaci.2004.06.032 15356552

[32] Prussin C Griffith DT Boesel KM Lin H Foster B Casale TB. Omalizumab treatment downregulates dendritic cell FcepsilonRI expression. J Allergy Clin Immunol. (2003) 112:1147–54. 10.1016/j.jaci.2003.10.003 14657874

[33] Maggi L Rossettini B Montaini G Matucci A Vultaggio A Mazzoni A Omalizumab dampens type 2 inflammation in a group of long-term treated asthma patients and detaches IgE from FcεRI. Eur J Immunol. (2018) 16:2005–14. 10.1002/eji.201847668 30252930

[34] Davies AM Allan EG Keeble AH Delgado J Cossins BP Mitropoulou AN. Allosteric mechanism of action of the therapeutic anti-IgE antibody omalizumab. J Biol Chem. (2017) 292:9975–87. 10.1074/jbc.M117.776476 28438838 PMC5473249

[35] Serrano-Candelas E Martinez-Aranguren R Valero A Bartra J Gastaminza G Goikoetxea MJ. Comparable actions of omalizumab on mast cells and basophils. Clin Exp Allergy (2016) 46:92–102. 10.1111/cea.12668 26509363

[36] Kim B Eggel A Tarchevskaya SS Vogel M Prinz H Jardetzky TS. Accelerated disassembly of IgE-receptor complexes by a disruptive macromolecular inhibitor. Nature (2012) 491:613–7. 10.1038/nature11546 23103871 PMC3504642

[37] Abdel-Gadir A Schneider L Casini A Charbonnier L-M Little SV Harrington T. Oral immunotherapy with omalizumab reverses the Th2 cell-like programme of regulatory T cells and restores their function. Clin Exp Allergy (2018) 48:825–36. 10.1111/cea.13161 29700872 PMC6021220

[38] Dantzer JA Wood RA. The use of omalizumab in allergen immunotherapy. Clin Exp Allergy (2018) 48:232–40. 10.1111/cea.13084 29315922

[39] Andorf S Purington N Block WM Long AJ Tupa D Brittain E. Anti-IgE treatment with oral immunotherapy in multifood allergic participants: a double-blind, randomised, controlled trial. Lancet Gastroenterol Hepatol. (2018) 3:85–94. 10.1016/S2468-1253(17)30392-8 29242014 PMC6944204

[40] Tsabouri S Tseretopoulou X Priftis K Ntzani EE. Omalizumab for the treatment of inadequately controlled allergic rhinitis: a systematic review and meta-analysis of randomized clinical trials. J Allergy Clin Immunol Pract. (2014) 2:332–40.e1. 10.1016/j.jaip.2014.02.001 24811026

[41] Masieri S Cavaliere C Begvarfaj E Rosati D Minni A. Effects of omalizumab therapy on allergic rhinitis: a pilot study. Eur Rev Med Pharmacol Sci. (2016) 20:5249–55. 28051241

[42] Holm JG Agner T Sand C Thomsen SF. Omalizumab for atopic dermatitis: case series and a systematic review of the literature. Int J Dermatol. (2017) 56:18–26. 10.1111/ijd.13353 27337170

[43] Kim DH Park KY Kim BJ Kim MN Mun SK. Anti-immunoglobulin E in the treatment of refractory atopic dermatitis. Clin Exp Dermatol. (2013) 38:496–500. 10.1111/j.1365-2230.2012.04438.x 23083013

[44] Corren J Shapiro G Reimann J Deniz Y Wong D Adelman D. Allergen skin tests and free IgE levels during reduction and cessation of omalizumab therapy. J Allergy Clin Immunol. (2008) 121:506–11. 10.1016/j.jaci.2007.11.026 18269927

[45] Molimard M Mala L Bourdeix I Le Gros V. Observational study in severe asthmatic patients after discontinuation of omalizumab for good asthma control. Respir Med. (2014) 108:571–6. 10.1016/j.rmed.2014.02.003 24565601

[46] Kopp MV Hamelmann E Bendiks M Zielen S Kamin W Bergmann K-C. Transient impact of omalizumab in pollen allergic patients undergoing specific immunotherapy. Pediatr Allergy Immunol. (2013) 24:427–33. 10.1111/pai.12098 23799935

[47] Kuehr J Brauburger J Zielen S Schauer U Kamin W Berg Von A. Efficacy of combination treatment with anti-IgE plus specific immunotherapy in polysensitized children and adolescents with seasonal allergic rhinitis. J Allergy Clin Immunol. (2002) 109:274–80. 10.1067/mai.2002.121949 11842297

[48] Braido F Corsico A Rogkakou A Ronzoni V Baiardini I Canonica GW. The relationship between allergen immunotherapy and omalizumab for treating asthma. Exp Rev Respir Med. (2015) 9:129–34. 10.1586/17476348.2015.1000866 25578528

[49] Chen M Land M. The current state of food allergy therapeutics. Hum Vaccin Immunother. (2017) 13:2434–42. 10.1080/21645515.2017.1359363 28846472 PMC5647972

[50] Tsabouri S Mavroudi A Feketea G Guibas GV Subcutaneous and sublingual immunotherapy in allergic asthma in children. Front Pediatric. (2017) 5:82 10.3389/fped.2017.00082 PMC5399038 28484690

[51] Passalacqua G Canonica GW Bagnasco D. Benefit of SLIT and SCIT for allergic rhinitis and asthma. Curr Allergy Asthma Rep. (2016) 16:88. 10.1007/s11882-016-0666-x 27957697

[52] Burks AW Calderon MA Casale T Cox L Demoly P Jutel M. Update on allergy immunotherapy: american academy of allergy, asthma & immunology/European academy of allergy and clinical immunology/PRACTALL consensus report. J Allergy Clin Immunol. (2013) 1288–96.e3. 10.1016/j.jaci.2013.01.049 23498595

[53] Akdis CA. Therapies for allergic inflammation: refining strategies to induce tolerance. Nat Med. (2012) 18:736–49. 10.1038/nm.2754 22561837

[54] Pitsios C Demoly P Bilò MB Gerth van Wijk R Pfaar O Sturm GJ. Clinical contraindications to allergen immunotherapy: an EAACI position paper. Allergy (2015) 70:897–909. 10.1111/all.12638 25913519

[55] Yukselen A. Allergen-specific immunotherapy in pediatric allergic asthma. Asia Pac Allergy (2016) 6:139–48. 10.5415/apallergy.2016.6.3.139 27489785 PMC4967613

[56] Schneider LC Rachid R LeBovidge J Blood E Mittal M Umetsu DT. A pilot study of omalizumab to facilitate rapid oral desensitization in high-risk peanut-allergic patients. J Allergy Clin Immunol. (2013) 132:1368–74. 10.1016/j.jaci.2013.09.046 24176117 PMC4405160

[57] Nadeau KC Schneider LC Hoyte L Borras I Umetsu DT. Rapid oral desensitization in combination with omalizumab therapy in patients with cow's milk allergy. J Allergy Clin Immunol. (2011) 127:1622–4. 10.1016/j.jaci.2011.04.009 21546071 PMC3396422

[58] Bégin P Dominguez T Wilson SP Bacal L Mehrotra A Kausch B. Phase 1 results of safety and tolerability in a rush oral immunotherapy protocol to multiple foods using Omalizumab. Allergy Asthma Clin Immunol. (2014) 10:7. 10.1186/1710-1492-10-7 24576338 PMC3936817

[59] Martorell-Calatayud C Michavila-Gómez A Martorell-Aragonés A Molini-Menchón N Cerdá-Mir JC Félix-Toledo R. Anti-IgE-assisted desensitization to egg and cow's milk in patients refractory to conventional oral immunotherapy. Pediatr Allergy Immunol. (2016) 27:544–6. 10.1111/pai.12567 27003835

[60] MacGinnitie AJ Rachid R Gragg H Little SV Lakin P Cianferoni A. Omalizumab facilitates rapid oral desensitization for peanut allergy. J Allergy Clin Immunol. (2017) 139:873–81.e8. 10.1016/j.jaci.2016.08.010 27609658 PMC5369605

[61] Balbino B Conde E Marichal T Starkl P Reber LL. Approaches to target IgE antibodies in allergic diseases. Pharmacol Ther. (2018) 191:50–64. 10.1016/j.pharmthera.2018.05.015 29909239

[62] Stumpp MT Binz HK Amstutz P. DARPins: a new generation of protein therapeutics. Drug Discov Today (2008) 13:695–701. 10.1016/j.drudis.2008.04.013 18621567

[63] Li J Mahajan A Tsai M-D. Ankyrin repeat: a unique motif mediating protein-protein interactions. Biochemistry (2006) 45:15168–78. 10.1021/bi062188q 17176038

[64] Binz HK Stumpp MT Forrer P Amstutz P Plückthun A. Designing repeat proteins: well-expressed, soluble and stable proteins from combinatorial libraries of consensus ankyrin repeat proteins. J Mol Biol. (2003) 332:489–503. 10.1016/S0022-2836(03)00896-9 12948497

[65] Plückthun A. Designed ankyrin repeat proteins (DARPins): binding proteins for research, diagnostics, and therapy. Annu Rev Pharmacol Toxicol. (2015) 55:489–511. 10.1146/annurev-pharmtox-010611-134654 25562645

[66] Eggel A Baumann MJ Amstutz P Stadler BM Vogel M. DARPins as bispecific receptor antagonists analyzed for immunoglobulin E receptor blockage. J Mol Biol. (2009) 393:598–607. 10.1016/j.jmb.2009.08.014 19683003

[67] Baumann MJ Eggel A Amstutz P Stadler BM Vogel M. DARPins against a functional IgE epitope. Immunol Lett. (2010) 133:78–84. 10.1016/j.imlet.2010.07.005 20673836

[68] Kambayashi T Koretzky GA. Proximal signaling events in Fc epsilon RI-mediated mast cell activation. J Allergy Clin Immunol. (2007) 119:544–52. 10.1016/j.jaci.2007.01.017 17336609

[69] Odom S Gomez G Kovarova M Furumoto Y Ryan JJ Wright HV. Negative regulation of immunoglobulin E-dependent allergic responses by Lyn kinase. J Exp Med. (2004) 199:1491–502. 10.1084/jem.20040382 15173205 PMC2211776

[70] Parravicini V Gadina M Kovarova M Odom S Gonzalez-Espinosa C Furumoto Y. Fyn kinase initiates complementary signals required for IgE-dependent mast cell degranulation. Nat Immunol. (2002) 3:741–8. 10.1038/ni817 12089510

[71] Gomez G Gonzalez-Espinosa C Odom S Baez G Cid ME Ryan JJ. Impaired FcεRI-dependent gene expression and defective eicosanoid and cytokine production as a consequence of Fyn deficiency in mast cells. J Immunol. (2005) 175:7602–10. 10.4049/jimmunol.175.11.7602 16301670

[72] Rivera J Cordero JR Furumoto Y Luciano-Montalvo C Gonzalez-Espinosa C Kovarova M. Macromolecular protein signaling complexes and mast cell responses: a view of the organization of IgE-dependent mast cell signaling. Mol Immunol. (2002) 38:1253–8. 10.1016/S0161-5890(02)00072-X 12217392

[73] Rosales C. Fcγ receptor heterogeneity in leukocyte functional responses. Front Immunol. (2017) 8:280. 10.3389/fimmu.2017.00280 28373871 PMC5357773

[74] Nimmerjahn F Ravetch JV. Fcgamma receptors as regulators of immune responses. Nat Rev Immunol. (2008) 8:34–47. 10.1038/nri2206 18064051

[75] Ono M Bolland S Tempst P Ravetch JV. Role of the inositol phosphatase SHIP in negative regulation of the immune system by the receptor Fc(gamma)RIIB. Nature (1996) 383:263–6. 10.1038/383263a0 8805703

[76] Ono M Okada H Bolland S Yanagi S Kurosaki T Ravetch JV. Deletion of SHIP or SHP-1 reveals two distinct pathways for inhibitory signaling. Cell (1997) 90:293–301. 9244303 10.1016/s0092-8674(00)80337-2

[77] Bolland S Ravetch JV. Inhibitory pathways triggered by ITIM-containing receptors. In: Dixon FJ editor. Advances in Immunology. New York, NY: Elsevier (1999). p. 149–77. 10.1016/s0065-2776(08)60019-x 10361574

[78] Kepley CL Taghavi S Mackay G Zhu D Morel PA Zhang K. Co-aggregation of FcgammaRII with FcepsilonRI on human mast cells inhibits antigen-induced secretion and involves SHIP-Grb2-Dok complexes. J Biol Chem. (2004) 279:35139–49. 10.1074/jbc.M404318200 15151996

[79] Cassard L Jönsson F Arnaud S Daëron M. Fcγ receptors inhibit mouse and human basophil activation. J Immunol. (2012) 189:2995–3006. 10.4049/jimmunol.1200968 22908332

[80] Cady CT Powell MS Harbeck RJ Giclas PC Murphy JR Katial RK. IgG antibodies produced during subcutaneous allergen immunotherapy mediate inhibition of basophil activation via a mechanism involving both FcgammaRIIA and FcgammaRIIB. Immunol Lett. (2010) 130:57–65. 10.1016/j.imlet.2009.12.001 20004689 PMC2849848

[81] Zhao W Kepley CL Morel PA Okumoto LM Fukuoka Y Schwartz LB. Fc gamma RIIa, not FcγRIIb, is constitutively and functionally expressed on skin-derived human mast cells. J Immunol. (2006) 177:694–701. 10.4049/jimmunol.177.1.694 16785568 PMC2176083

[82] Sellge G Barkowsky M Kramer S Gebhardt T Sander LE Lorentz A. Interferon-γ regulates growth and controls Fcγ receptor expression and activation in human intestinal mast cells. BMC Immunol. (2014) 15:27. 10.1186/1471-2172-15-27 24996251 PMC4227132

[83] Daëron M Malbec O Latour S Arock M Fridman WH. Regulation of high-affinity IgE receptor-mediated mast cell activation by murine low-affinity IgG receptors. J Clin Invest. (1995) 95:577–85. 10.1172/JCI117701 7860741 PMC295517

[84] Tam SW Demissie S Thomas D Daëron M. A bispecific antibody against human IgE and human FcgammaRII that inhibits antigen-induced histamine release by human mast cells and basophils. Allergy (2004) 59:772–80. 10.1111/j.1398-9995.2004.00332.x 15180766

[85] Strait RT Morris SC Finkelman FD. IgG-blocking antibodies inhibit IgE-mediated anaphylaxis *in vivo* through both antigen interception and Fc gamma RIIb cross-linking. J Clin Invest. (2006) 116:833–41. 10.1172/JCI25575 16498503 PMC1378186

[86] Zhu D Kepley CL Zhang M Zhang K Saxon A. A novel human immunoglobulin Fc gamma Fc epsilon bifunctional fusion protein inhibits Fc epsilon RI-mediated degranulation. Nat Med. (2002) 8:518–21. 10.1038/nm0502-518 11984598 PMC1866216

[87] Siraganian RP Zhang J Suzuki K Sada K. Protein tyrosine kinase Syk in mast cell signaling. Mol Immunol. (2002) 38:1229–33. 10.1016/S0161-5890(02)00068-8 12217388

[88] Costello PS Turner M Walters AE Cunningham CN Bauer PH Downward J Tybulewicz VL. Critical role for the tyrosine kinase Syk in signalling through the high affinity IgE receptor of mast cells. Oncogene (1996) 13:2595–605. 9000133

[89] Mertsching E Bafetti L Hess H Perper S Giza K Allen LC. A mouse Fcgamma-Fcepsilon protein that inhibits mast cells through activation of FcgammaRIIB, SH2 domain-containing inositol phosphatase 1, and SH2 domain-containing protein tyrosine phosphatases. J Allergy Clin Immunol. (2008) 121:441–7.e5. 10.1016/j.jaci.2007.08.051 17949802

[90] Cemerski S Chu SY Moore GL Muchhal US Desjarlais JR Szymkowski DE. Suppression of mast cell degranulation through a dual-targeting tandem IgE-IgG Fc domain biologic engineered to bind with high affinity to FcγRIIb. Immunol Lett. (2012) 143:34–43. 10.1016/j.imlet.2012.01.008 22305932

[91] Chu SY Vostiar I Karki S Moore GL Lazar GA Pong E. Inhibition of B cell receptor-mediated activation of primary human B cells by coengagement of CD19 and FcgammaRIIb with Fc-engineered antibodies. Mol Immunol. (2008) 45:3926–33. 10.1016/j.molimm.2008.06.027 18691763

[92] Moore GL Chen H Karki S Lazar GA. Engineered Fc variant antibodies with enhanced ability to recruit complement and mediate effector functions. MAbs (2010) 2:181–9. 10.4161/mabs.2.2.11158 20150767 PMC2840237

[93] Zhang K Kepley CL Terada T Zhu D Perez H Saxon A. Inhibition of allergen-specific IgE reactivity by a human Ig Fcgamma-Fcepsilon bifunctional fusion protein. J Allergy Clin Immunol. (2004) 114:321–7. 10.1016/j.jaci.2004.03.058 15316510

[94] Chu SY Horton HM Pong E Leung IWL Chen H Nguyen D-H. Reduction of total IgE by targeted coengagement of IgE B-cell receptor and FcγRIIb with Fc-engineered antibody. J Allergy Clin Immunol. (2012) 129:1102–15. 10.1016/j.jaci.2011.11.029 22257644

[95] Eggel A Buschor P Baumann MJ Amstutz P Stadler BM Vogel M. Inhibition of ongoing allergic reactions using a novel anti-IgE DARPin-Fc fusion protein. Allergy (2011) 66:961–8. 10.1111/j.1398-9995.2011.02546.x 21272035

[96] Buschor P Eggel A Zellweger F Stadler BM Vogel M. Improved FcγRIIb targeting functionally translates into enhanced inhibition of basophil activation. Int Arch Allergy Immunol (2014) 163:206–14. 10.1159/000358487 24557487

[97] Zellweger F Gasser P Brigger D Buschor P Vogel M Eggel A. A novel bispecific DARPin targeting FcγRIIB and FcεRI-bound IgE inhibits allergic responses. Allergy (2017) 72:1174–83. 10.1111/all.13109 27997998

